# Enhancing anti-tumor immunity through local gene delivery to lymph nodes

**DOI:** 10.1186/2051-1426-3-S2-P431

**Published:** 2015-11-04

**Authors:** Neil M Dold, Christopher M Jewell

**Affiliations:** 1 Fischell Department of Bioengineering, University of Maryland - College Park, College Park, MD, USA

## Introduction

Biodegradable polymer carriers offer attractive features for therapeutic cancer vaccines including delivery of multiple vaccine components, efficient internalization, and sustained release of adjuvants and tumor-associated antigens (TAAs). We previously demonstrated that local delivery of depots containing nucleic acid-based toll-liked receptor agonists (TLRas) to lymph nodes (LNs) potently enhances antigen-specific T cell immunity. Building on this work, we hypothesized that local LN delivery of microparticles loaded with TAA-encoding plasmid DNA (pDNA) and TLRas might drive strong local expression and presentation of antigen by LN-resident antigen presenting cells. These effects could help drive more potent and effective CD8+ T cell functions that slow or stop tumor growth.

## Methods

Plasmids were designed and synthesized to encode model reporter genes or a conserved T cell peptide epitope, TRP2 (SVYDFFVWL) for human and murine melanoma. Screening studies with primary dendritic cells utilized poly(lactic-*co*-glycolide) microparticles synthesized by water-oil-water emulsion, stabilized with polyvinyl alcohol. The inner aqueous phase was loaded with a TLR9 agonist (CpG oligodeoxynucleotide) and pDNA. Size and loading were characterized by laser diffraction and UV-vis spectroscopy. Murine splenocytes expressing CD11c were harvested, isolated, and treated overnight in triplicate with empty particles or particles encapsulating CpG only, pDNA only, or both CpG+pDNA. Cells were analyzed by flow cytometry using markers for viability (DAPI-) and dendritic cell activation (CD40+, CD80+, CD86+).

## Results

The pDNA vector for the TRP2 epitope was designed to express degradable peptide spacers (AAY) flanking repeating units of the antigenic epitope. This vector was synthesized with and without a sequence encoding ubiquitin to provide efficient proteasomal peptide processing and antigen expression. Particles exhibited mean diameters 2.5-4.4µm depending on cargo and synthesis energy. Cargo loading was measured for CpG particles at 3.8µg CpG/mg particles and for pDNA particles at 11.1µg pDNA/mg particles. During *ex vivo* screening, particles containing cargo increased CD40, CD80, and CD86 expression compared to empty particles and did not impact cell viability compared to controls treated with soluble TLRas.

## Discussion

Initial studies demonstrate microparticles loaded with TLRa and pDNA effectively stimulate primary immune cells, supporting a platform for loading and driving local TAA expression alongside vaccine components like TLRas. Ongoing studies will assess gene expression levels in primary cells and secondary lymph organs using a mouse melanoma model and will correlate local TAA expression with antigen-specific T cell expansion and efficacy during tumor challenge.

